# Changes of synaptic vesicles in three-dimensional synapse models by treatment with umbelliferone in scopolamine-induced hippocampal injury model

**DOI:** 10.1186/s42649-024-00095-y

**Published:** 2024-01-23

**Authors:** Ga-Young Choi, Eunyoung Moon, Hyosung Choi, Hee-Seok Kweon

**Affiliations:** Center of Research Equipment, Korea Basic Science Institute, Cheongju, 28119 Republic of Korea

**Keywords:** Umbelliferone, Scopolamine, Synaptic vesicle, Hippocampus, Electron tomography, High voltage electron microscopy

## Abstract

The neuroprotective effects of umbelliferone (UMB) were visualized in three-dimensional (3D) images on vesicle density changes of organotypic hippocampal slice tissues (OHSCs) induced by scopolamine by high voltage electron microscopy. Observations revealed that the number of vesicles decreased in OHSCs induced by scopolamine, and UMB was found to inhibit scopolamine-induced reduction in vesicles, resulting in an increase in vesicle count. These 3D models provide valuable insight for understanding the increase of synapse vesicles in hippocampal tissues treated with UMB.

## Description

Umbelliferone (UMB) is a 7-hydroxycoumarin, which is a pharmacologically active component derived from the Apiaceae family. Anti-inflammatory, antioxidant (Althunibat et al. 2022), anti-glycation, antidiabetic (Jin and Chen 2022), anticancer (Aslanturk and Askin Celik 2023), and anti-hepatic (Hassanein et al. 2021) properties have been reported from UMB. However, the neuroprotective effects of UMB have not been confirmed by ultrastructural morphological approaches. In previous studies, the neuroprotective effects of UMB were investigated in an animal model where learning and memory were impaired using scopolamine (SCOP), a non-selective muscarinic acetylcholine receptor antagonist (Wan et al. 2019). This investigation involved behavioral experiments, molecular biology analysis, and electrophysiological analysis (Choi et al. 2023). In this study, we visualized and analyzed the neuroprotective effects of UMB treatment on hippocampal synaptic ultrastructures in organotypic hippocampal slice cultures using a biological high voltage electron microscopy (Bio-HVEM) system.

The organotypic hippocampal slice tissues used in this study followed the protocol described in the previous study (Stoppini et al. 1991). The hippocampus was extracted from the rat’s brain and immersed in HBSS containing 20 mM HEPES. The hippocampus was chopped into 350 μm-thick slices using a chopper (Cavey Laboratory Engineering Co., Guildford, Surrey, UK) and placed on a 0.4 µm membrane insert (Millicell-CM; Millipore®, Merck KGaA, Darmstadt, Germany) in a six-well tissue culture plate with a medium. The tissues were treated with control, SCOP (300 μM), or UMB (10 μM) + SCOP (300 μΜ) and postfixed in 2% OsO_4_/1.5% potassium ferrocyanide for 1 h. The tissues were submerged in 1% thiocarbohydrazide (Ted Pella Inc., Redding, CA, USA) for 20 min, followed by 2% OsO_4_ for 30 min. Next, the tissues were incubated overnight in 1% uranyl acetate at 4 °C, followed by a lead aspartate solution at 60 °C for 30 min.

For high voltage electron tomography, the tissues were dehydrated in a series of ethanol and embedded in Epon 812 resin, and relatively 300 nm-thick sections were obtained on the grids. The grids were placed in a double-tilt specimen holder (EM-Z19103TDTH; JEOL Ltd., Tokyo, Japan) and imaged in the KBSI Bio-HVEM System (JEM-1000BEF; JEOL Ltd., Tokyo, Japan) operating at 1 MeV. A total of 121 tilt images were captured by transmission electron microscopy (TEM) recorder software (JEOL Ltd., Tokyo, Japan). The digitized tilting images were aligned and tomographically reconstructed using Composer and Visualizer-Kai software (TEMography.com, System In Frontier Inc., Tokyo, Japan). Selected tomograms were manually segmented, and AMIRA software (Thermo Fisher Scientific Inc., Waltham, MA, USA) was used for volume rendering and 3D modeling (Kim et al. 2012).

**Fig. 1:**
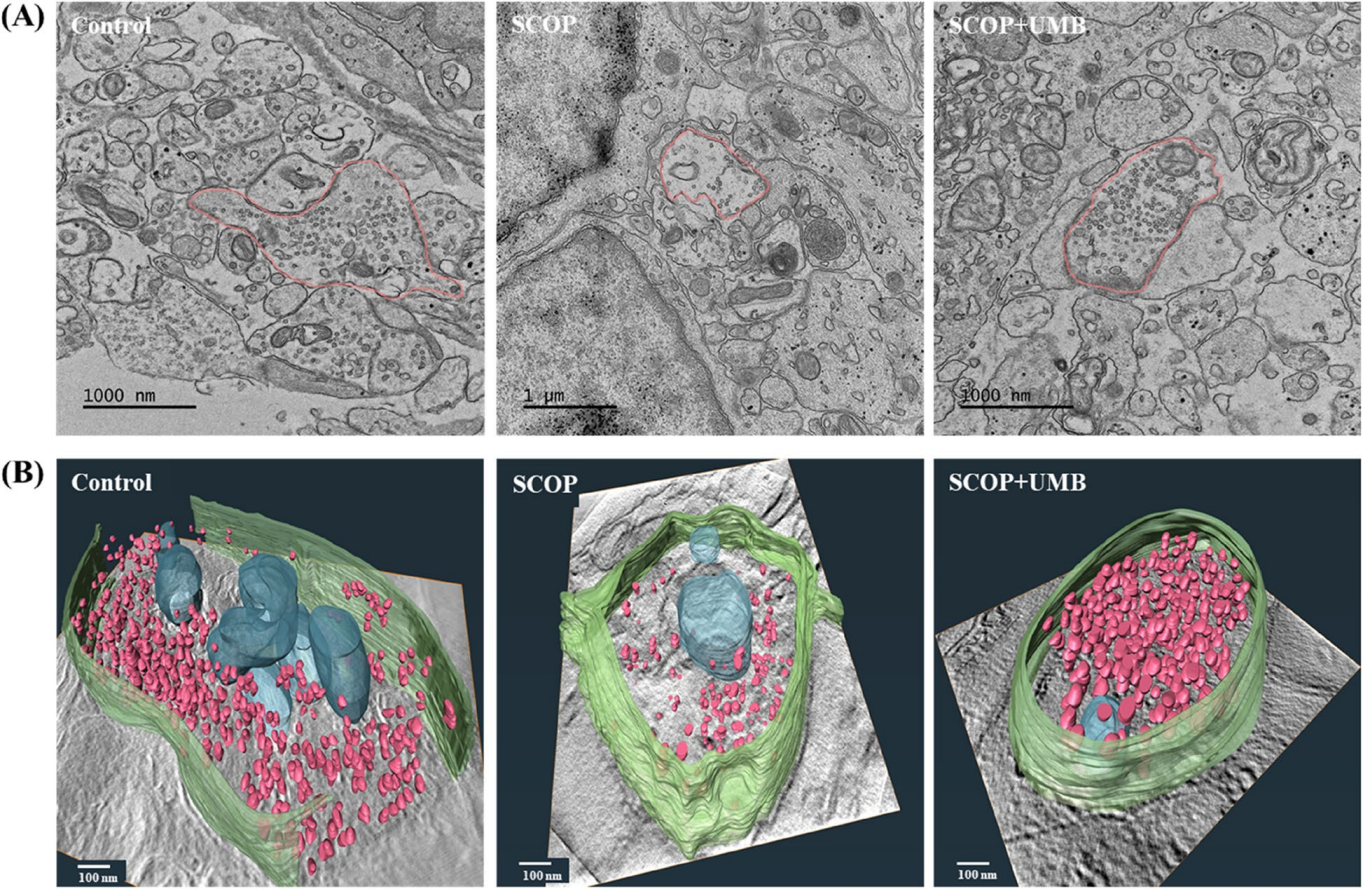
Hippocampal synaptic ultrastructure of the UMB-treated OHSC tissues. **A** Representative 2D electron micrograph images of synapses in the hippocampus of each group (control, SCOP, SCOP + UMB). Scale bar = 1000 nm. **B** 3D models of the reconstructed synapse in each group showing synaptic vesicles (red) and mitochondria (blue) on synaptic ultrastructure of the hippocampus using the KBSI Bio-HVEM System. Scale bar = 100 nm

As shown in Fig. 1, the synaptic vesicle density decreased in the SCOP group compared to the control group. The SCOP + UMB group, in contrast, reversed the reduction in synaptic vesicle density observed in the SCOP group. In particular, the number of synaptic vesicles within 300 nm of the active zone increased dramatically in the SCOP + UMB group compared to the SCOP group. Increased synaptic vesicle density is strongly associated with learning and memory improvement due to changes in synaptic morphology and structure (Sousa et al. 2000; Lee et al. 2018). Therefore, our results demonstrate that UMB mitigates SCOP-induced learning and memory impairments. In conclusion, through the 3D models which make volumetric analysis easier than 2D, we can clearly suggest that UMB may be a crucial neuroprotective compound for enhancing learning and memory in neurodegenerative diseases.

## Data Availability

Not applicable. “Please contact the corresponding author for data requests.”
